# Intraocular lens power calculation for plus and minus lenses in high myopia using partial coherence interferometry

**DOI:** 10.1007/s10792-020-01684-y

**Published:** 2021-02-01

**Authors:** Matthias Fuest, Niklas Plange, David Kuerten, Hannah Schellhase, Babac A. E. Mazinani, Peter Walter, Stephan Kohnen, Randolf A. Widder, Gernot Roessler

**Affiliations:** 1 Department of Ophthalmology, RWTH Aachen University, Pauwelsstrasse 30, 52074 Aachen, Germany; 2 ACD Augen Centrum Dreiländereck, Aachen, Germany; 3 Department of Ophthalmology, St. Martinus-Krankenhaus Düsseldorf, Düsseldorf, Germany; 4 Department of Ophthalmology, University of Cologne, Cologne, Germany

**Keywords:** Cataract, Biometry, Pathologic, Myopia, IOL, Calculation

## Abstract

**Purpose:**

We assessed the accuracy of lens power calculation in highly myopic patients implanting plus and minus intraocular lenses (IOL).

**Methods:**

We included 58 consecutive, myopic eyes with an axial length (AL) > 26.0 mm, undergoing phacoemulsification and IOL implantation following biometry using the IOLMaster 500. For lens power calculation, the Haigis formula was used in all cases. For comparison, refraction was back-calculated using the Barrett Universal II (Barrett), Holladay I, Hill-RBF (RBF) and SRK/T formulae.

**Results:**

The mean axial length was 30.17 ± 2.67 mm. Barrett (80%), Haigis (87%) and RBF (82%) showed comparable numbers of IOLs within 1 diopter (D) of target refraction. Visual acuity (BSCVA) improved (*p* < 0.001) from 0.60 ± 0.35 to 0.29 ± 0.29 logMAR (> 28-days postsurgery). The median absolute error (MedAE) of Barrett 0.49 D, Haigis 0.38, RBF 0.44 and SRK/T 0.44 did not differ. The MedAE of Haigis was significantly smaller than Holladay (0.75 D; *p* = 0.01). All median postoperative refractive errors (MedRE) differed significantly with the exception of Haigis to SRK/T (*p* = 0.6): Barrett − 0.33 D, Haigis 0.25, Holladay 0.63, RBF 0.04 and SRK/T 0.13. Barrett, Haigis, Holladay and RBF showed a tendency for higher MedAEs in their minus compared to plus IOLs, which only reached significance for SRK/T (*p* = 0.001). Barrett (*p* < 0.001) and RBF (*p* = 0.04) showed myopic, SRK/T (*p* = 002) a hyperopic shift in their minus IOLs.

**Conclusions:**

In highly myopic patients, the accuracies of Barrett, Haigis and RBF were comparable with a tendency for higher MedAEs in minus IOLs. Barrett and RBF showed myopic, SRK/T a hyperopic shift in their minus IOLs.

## Introduction

Deviation from target refraction is one of the most frequent indications for secondary intervention following the implantation of foldable intraocular lenses (IOL), excluding posterior capsule opacification [1]. In addition to postoperative anterior chamber depth and effective lens position (ELP), preoperative axial length (AL) measurement represents the most important error source for incorrect IOL power prediction, particularly in myopic eyes [2, 3]. The unaided increase of visual acuity is a main target of cataract surgery, particularly in case of clear lens exchange [4]. Besides the increase in visual acuity, the possibility of myopia reduction can further increase patient satisfaction [5]. Partial coherence laser interferometry (PCI) yields AL measurements ten times more accurate than ultrasound [6]. However, it can be limited in cases of retinal detachment, fixation problems or dense cataract [7, 8].

Generations of formulae have been proposed since the 1970s, differing mainly in the way they estimate the ELP [9, 10]. Moving from regression-based formulae to theoretical formulae helped to further increase accuracy as these third-generation formulae now used biometric data to estimate the effective lens position within the eye (i.e. SRK/T (T = theoretical), Holladay I, Hoffer Q). Fourth-generation formulae include additional parameters to calculate the ELP (Haigis- preoperative anterior chamber depth (ACD), Olsen-ACD and lens thickness). The Barrett universal II formula (Barrett) uses a theoretical model eye, in which anterior chamber depth is related to axial length and keratometry. The formula was described as universal because it is supposed to work for different lens styles and for eyes with short, medium, and long axial lengths [11, 12]. The Hill-RBF (radial basis function; RBF) formula is a big-data/neural-net-based formula, incorporating data from thousands of eyes. It does not rely on a specific equation but evaluates existing data to predict results for new sets of measurements 12].

Previous studies have focused on the use of different formulae for biometry in highly myopic eyes [2, 3, 10, 13, 14]. However, studies investigating the specific effects of minus and plus IOLs are rare, particularly for the new formulae (Barrett and RBF).

In this study, we determined the median absolute (MedAE) and the median refractive error (MedRE) of phacoemulsification and IOL implantation following optical biometry using the Barrett, Haigis, Holladay I, RBF and SRK/T formulae, particularly comparing the differential effects of minus and plus IOLs in patients with highly myopic eyes.

## Methods

In a retrospective trial, we included a series of 58 consecutive highly myopic eyes, 31 right and 27 left, of 38 patients, 21 females, with an axial length (AL) > 26.0 mm, which underwent uncomplicated phacoemulsification and posterior chamber IOL implantation following biometry using the Zeiss IOLMaster 500 (Version 7.5.3.0084, Carl Zeiss Meditec, Jena, Germany). Patients with ocular abnormalities complicating PCI, such as multiple prior ocular surgery, endotamponades, corneal alterations, e.g. scars, or abnormalities of the posterior pole, e.g. macular holes, oedema were excluded. For lens power calculation, the Haigis formula was used in all cases. In addition, IOL power predictions were back-calculated using the Barrett Universal II (Barrett; https://www.apacrs.org/barrett_universal2, Version 1.05) Holladay I, Hill-RBF (RBF; https://rbfcalculator.com, Version 2.0) and SRK/T formulae.

All patients underwent phacoemulsification and insertion of an acrylic posterior chamber IOL into the capsular bag. Operations were performed uneventfully by one experienced surgeon (NP) at the Department of Ophthalmology at the RWTH Aachen University, Germany. The implanted IOLs were the single piece AcriTec 44S, later branded as CT Spheris 204 (Carl Zeiss Meditec, 58 eyes, IOL power range − 10.0–30.0 D, IOLs in the study − 10.0–14.5 D).

Generally, IOLs aiming for a mild or moderate postoperative myopia were chosen. However, in some cases such as large anisometropia, IOLs with a higher targeted postoperative myopia were implanted.

The User Group for Laser Interference Biometry (ULIB) optimized constants of the IOL and for Barrett and RBF the optimized SRK/T A-constant, as instructed on the according websites, were used (http://ocusoft.de/ulib, date of access 18 July 2019).

The constants were: nominal: 118.0 / Haigis: a0 = 0.93; a1 = 0.40; a2 = 0.10 / Holladay I: sf = 1.40 / SRKT: A = 118.3.

To determine the MedAE and the MedRE, the postoperative spherical equivalent refractive error was recorded at least > 28 days after surgery by subjective refraction. Patients that underwent ocular surgery during the follow-up period were excluded. IOLMaster examination for IOL calculation recorded AL-measurement, keratometry and ACD. Only measurements with a signal-to-noise ratio of at least 2.0 were included [15]. Data on age, sex, ocular history and visual acuity were also collected. Pre- and postoperative refractive errors were measured by autorefraction (AR-1; Version AR18V10101, Oculus, Wetzlar, Germany) followed by subjective refraction. MedAEs and refractive shifts were calculated for all formulae. Visual acuity was tested with the optimized subjective refractive correction, using a 5 m projected Snellen chart. Visual acuity values were converted to logMAR for descriptive purposes.

## Statistical analysis

Statistical analysis was performed using Software Stat View for Windows (Version 5.0; SAS Institute Inc., http://www.sas.com).

Patient characteristics were expressed as the mean ± standard deviation (range: min to max). As the absolute and refractive errors do not follow a normal Gaussian distribution [16], values were expressed as the median (95% confidence interval (CI)) and compared by Mann–Whitney-U or Wilcoxon signed-rank test. The Bonferroni correction was used for multiple comparisons. A *p* value of less than 0.05 was considered statistically significant.

## Results

### Overall

Mean age of all patients was 61.5 ± 11.4 (range: 41–79) years. Preoperative spherical equivalent (SE) could not reliably be evaluated in some of the highly myopic patients. In 52 eyes, it was − 15.92 ± 6.66 (range: − 28.88 − 4.5) diopters (D).

Mean AL measured by IOL Master was 30.18 ± 2.67 (range: 26.07–35.90) mm. Mean preoperative best spherical corrected visual acuity (BSCVA) was 0.60 ± 0.35 (range: 1.3–0.0) logMAR. Mean ACD was 3.39 ± 0.50 (range: 2.1–4.1) mm.

Looking at all ALs, IOLs were aimed at a postoperative refraction of − 0.63 ± 1.05 (range: − 2.84–1.88) D (Barrett), − 1.25 ± 0.99 (range: − 3.5–0.75) D (Haigis), − 1.64 ± 0.98 (range: − 4.0–0.25) D (Holladay), − 0.98 ± 0.96 (range: − 3.17–1.30) D (RBF) and − 0.98 ± 0.96 (range: − 4.5–1.0) D (SRK/T). The mean implanted IOL power was 3.4 ± 6.0 (range: − 10.0–14.5) D.

At the follow-up visit (> 28-day postsurgery), BSCVA had improved (*p* < 0.001)) to 0.29 ± 0.29 (range: 0.0–1.3) logMAR. MedRE was − 0.33 D (CI: − 0.57– − 0.12) for Barrett, 0.25 (CI: 0.10–0.50) for Haigis, 0.63 (CI: 0.38–0.88) for Holladay, 0.04 (CI: − 0.22–0.25) for RBF and 0.13 (CI: − 0.10–0.47) for SRK/T (Table 1). All MedREs differed significantly (*p* < 0.01), with the exception of Haigis to SRK/T (*p* = 0.7).

**Table 1:** Differences in median absolute error (MedAE) and median refractive error (MedRE) with their 95% confidence intervals (CI) in dioptres (D) for all plus and minus IOLs. Significant p-values (< 0.05) are shown in bold numbers

Formula	Plus IOL		Minus IOL		Minus vs. Plus *(p* =*)*	All IOLs		Plus IOL		Minus IOL		Minus vs. Plus *(p* =*)*	All IOLs	
	MedAE (CI)	*n* =	MedAE ± (CI)	*n* =	MedAE	MedAE (CI)	n =	MedRE ± (CI)	*n* =	MedRE (CI)	*n* =	MedRE	MedRE (CI)	*n* =
Barrett	0.44 (0.29 – 0.63	44	0.61 (0.13 – 1.38)	13	0.1	0.49 (0.34 – 0.64)	57	− 0.22 (− 0.39 – − 0.02)	44	− 1.0 (− 1.56 – − 0.44)	13	** < 0.001**	− 0.33 (− 0.57 – − 0.12)	57
Haigis	0.25 (0.25 – 0.50)	44	0.63 (0.22 – 1.01)	13	0.2	0.38 (0.25 – 0.63)	57	0.25 (0.03 – 0.50)	44	0.25 (− 0.68 – 0.64)	13	0.53	0.25 (0.10 – 0.50)	57
Holladay	0.63 (0.38 – 0.92)	45	1.13 (0.36 – 1.50)	13	0.1	0.75 (0.40 – 1.00)	58	0.63 (0.25 – 0.88)	45	1.00 (0.07 – 1.39)	13	0.38	0.63 (0.38 – 0.88)	58
RBF	0.42 (0.25 – 0.52)	44	0.94 (0.04 – 1.90)	9	0.1	0.44 (0.26 – 0.55)	53	0.08 (− 0.17 – 0.41)	44	− 0.94 (− 1.89 – 0.57)	9	**0.04**	0.04 (− 0.22 – 0.25)	53
SRK/T	0.38 (0.25 – 0.63)	45	1.50 (0.46 – 1.89)	13	**0.001**	0.44 (0.25 – 0.75)	58	0.13 (− 0.13 – 0.30)	45	1.50 (− 0.13 – 1.89)	13	**0.002**	0.13 (− 0.10 – 0.47)	58

**Fig. 1:**
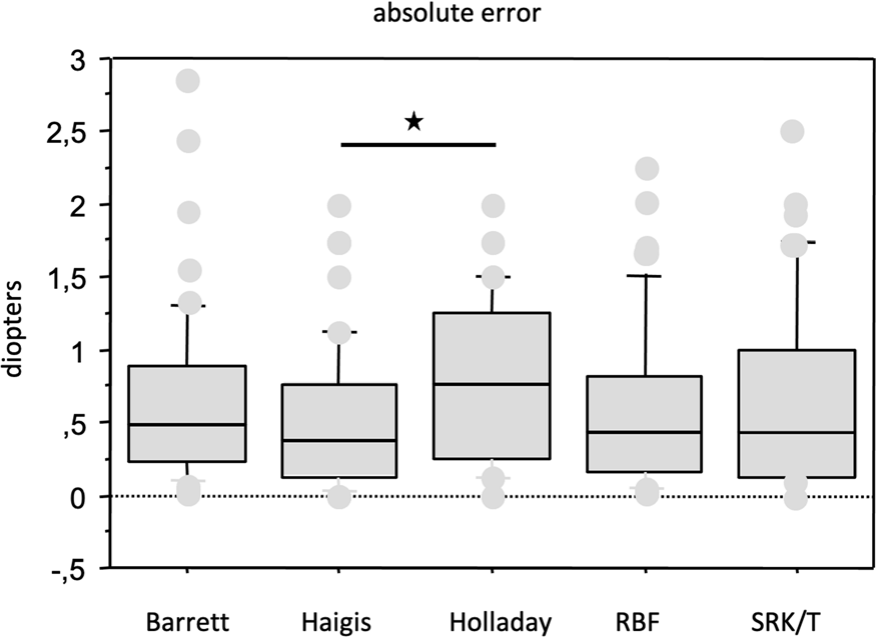
Median absolute error (MedAE) for all implanted IOLs. The MedAE of Haigis was significantly smaller than Holladay (*p* < 0.001, *). The differences between the other MedAEs did not reach significance

The MedAEs for all IOLs were Barrett 0.49 D (CI: 0.34 – 0.64), Haigis 0.38 (CI: 0.25–0.63), Holladay 0.75 (CI: 0.40–1.13), RBF 0.44 (CI: 0.25–0.57) and SRK/T 0.44 (CI: 0.25–0.75) (Table 1). The MedAE of Haigis was significantly smaller than Holladay (*p* < 0.001, Fig. 1). The differences between the other MedAEs did not reach significance.

Number and percentage of eyes deviating from the intended refraction by an absolute error ≤ 0.5 D, ≤ 1.0 D and > 1.0 D for all implanted IOLs are displayed in Table 2.

**Table 2:** Number and percentage of eyes deviating from the intended refraction by a median absolute error (MedAE) ≤ 0.5 D, ≤ 1.0 D and > 1.0 D for all implanted IOLs

Formula	MedAE *n* = (%)			
	≤ 0.5 D	≤ 1.0 D	> 1.0 D	Total
Barrett	30 (53%)	46 (81%)	11 (19%)	57 (100%)
Haigis	36 (63%)	50 (88%)	7 (12%)	57 (100%)
Holladay	23 (40%)	38 (66%)	20 (34%)	58 (100%)
RBF	31 (58%)	44 (83%)	9 (17%)	53 (100%)
SRK/T	32 (55%)	44 (76%)	14 (24%)	58 (100%)

### Minus vs. plus

The ALs of the minus (33.91 ± 1.71 mm) and plus (29.10 ± 1.76 mm) IOL groups differed significantly (*p* < 0.001). The minus IOL group (− 25.10 ± 2.37 D) was significantly (*p* < 0.001) more myopic before surgery than the plus IOL (− 13.99 ± 5.57 D) group.

**Fig. 2:**
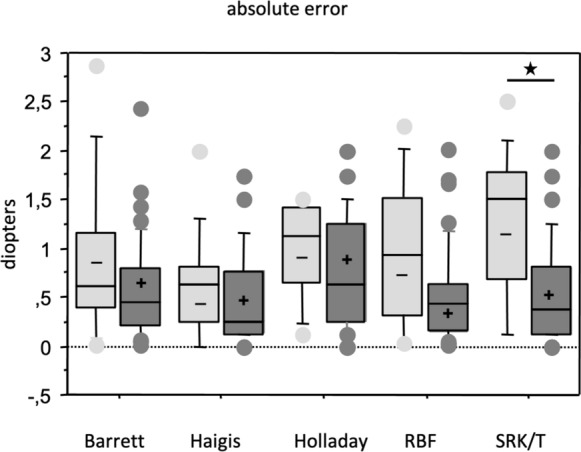
Median absolute error (MedAE) for plus and minus IOLs. Barrett, Haigis, Holladay and RBF showed a tendency for higher MedAEs in their minus compared to plus IOLs, which only reached significance for SRK/T (*p* = 0.001, *)

Differences in MedAEs and MedREs for plus and minus IOLs within one formula are displayed in Table 1. Barrett, Haigis, Holladay and RBF showed a tendency for higher MedAEs in their minus compared to plus IOLs, which only reached significance for SRK/T (*p* = 0.001) (Fig. 2).

**Fig. 3:**
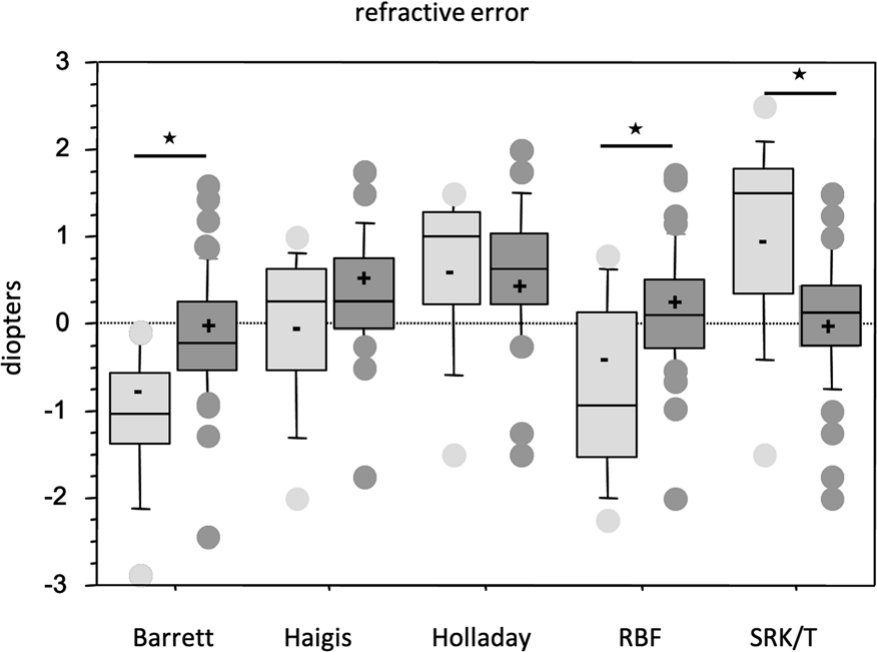
Median refractive error (MedRE) for plus and minus IOLs. Barrett (*p* < 0.001) and RBF (*p* = 0.04) showed myopic, SRK/T (*p* = 0.002) a hyperopic shift in their minus IOLs

Barrett (< 0.001) and RBF (*p* = 0.04) showed myopic, SRK/T (*p* = 0.002) a hyperopic shift in their minus IOLs (Table 1, Fig. 3).

Comparing the MedAEs of only the plus IOLs between formulae, RBF (*p* = 0.002), Haigis (*p* < 0.001) and SRK/T (*p* = 0.004) did not differ but were significantly smaller than Holladay.

Comparing the MedAEs of only the minus IOLs between formulae, Holladay (*p* = 0.01) and Haigis (*p* = 0.02) had a significantly smaller MedAE than SRK/T. The differences between other formulae did not reach significance.

Looking at the MedREs of only the plus IOLs between formulae, all MedREs differed significantly (*p* < 0.001) apart from RBF vs. SRK/T (*p* = 0.38). In minus IOLs, all MedREs differed significantly between formulae (*p* < 0.05).

## Discussion

To investigate the refractive outcomes of cataract surgery, different approaches are possible. To evaluate the accuracy of the formula itself,

“the mean error (ME) of the study group for each formula should be made to equal zero by changing the lens factor (constant) individually for each formula.” [10, 16].

The formulae can be optimized for each centre, as was previously suggested by Wang and Koch (WK) in 2011 [3].

However, this approach requires large sample sizes and individual calculations, which might result challenging for regular cataract surgery clinics.

We therefore analysed the refractive outcomes relying on the ULIB optimized IOL constants (http://ocusoft.de/ulib), to raise awareness for the disparities between formulae, and particularly between minus and plus IOLs, when using these constants.

In our analysis of the refractive outcome of 63 highly myopic eyes undergoing phacoemulsification and IOL implantation, we found that overall the accuracies indicated by MedAE of Barrett, Haigis and RBF were high and comparable. The formulae rendered different MedREs from more myopic outcomes (Barrett) to almost emmetropia (Haigis, RBF and SRK/T) to hyperopic outcomes (Holladay). Looking at differences within the formulae between minus and plus IOLs, we found that Barrett, Haigis, Holladay and RBF showed a tendency for higher MedAEs in their minus IOLs, which was significant for SRK/T. Barrett and RBF showed myopic, SRK/T a hyperopic shift in their minus IOLs.

Postoperative ACD and ELP as well as preoperative AL determination represent important possible error sources for incorrect IOL power calculation [17, 18]. However, the ACD as a parameter for postoperative ELP loses relevance with increasing AL, as the refraction change per mm IOL deviation was shown to be three times higher in short compared to long eyes with an AL beyond 27 mm [19]. Consequently, most studies focus on AL measurement and IOL power calculation formulae as the most crucial error sources in highly myopic eyes [2, 4, 10, 18].

Nevertheless, PCI measurements depend on the signal reflection of the retinal pigment epithelium. With larger AL values, the prevalence of morphological alterations like rarefication of Bruch’s membrane and the pigment epithelium increase leading to reduced measurement quality. [2, 20] This might be a cofactor responsible for the tendency for higher MedAEs we found in minus compared to plus IOLs.

Different approaches have been investigated to minimize the MedAE in highly myopic eyes. Haigis et. al. previously suggested that optimized IOL constants should be used [19]. However, optimizing the constants individually for minus and plus IOLs is difficult to realize in a clinical setup. In addition, the adjustment of AL for intraocular lens power calculation in eyes with ALs above 25.0 mm suggested by WK[3] has shown to be centre and lens specific and one of the largest retrospective case series on 13,301 cataract surgeries by Melles et al. even found worse outcomes, for example Haigis, when the AL was optimized according to WK [10].

In our opinion, if optimization is not possible, improving the knowledge on the specific refractive and absolute errors for individual formulae and constants can be helpful.

The great majority of previous studies evaluating the refractive outcomes of highly myopic eyes following cataract surgery focused on IOLs by Alcon (Fort Worth, USA) [2, 3, 10, 14, 19, 21]. As IOLs differ in their refractive outcomes [10], studies on other IOL suppliers are helpful for the community.

In our analysis, the fourth-generation formulae Barrett, Haigis and RBF showed high and comparable accuracies, while third-generation formulae Holladay and SRK/T performed worse, which was shown before. [2, 10, 13] However, third-generation formulae remain important and popular. They balance relatively good results with simplicity because the only biometric data points required are keratometry and axial length [9, 10].

The newer formulae also showed specific alterations, which have to be considered. The changes in IOL geometry of low- and negative-power IOLs create the potential for inaccurate IOL calculation in long eyes [14, 19]. Accordingly, our data replicated larger MedAEs in minus IOLs compared to plus IOLs. A clear additional hyperopic refractive error when minus lenses were used in comparison with plus lenses, as it was previously reported by Haigis et al., [19] was only found for SRK/T. On the other hand, Barrett and RBF showed myopic shifts in minus IOLs, while the MedRE with Haigis did not differ significantly between plus and minus IOLs.

In this study, we analysed formulae integrated in the IOLMaster 500. In addition, the RBF calculator is openly available (https://rbfcalculator.com). On the website, usage is particularly recommended for the IOL models SN60WF and MA60MA by Alcon and biconvex and meniscus IOL models within the power range of − 5.00– + 30.00 dioptres. IOLs for ALs larger than 35.0 mm cannot be calculated. This affected three eyes in our dataset. Also the calculator is believed to work best with targeted emmetropic refractions [10].

The Barrett Universal II calculator is also openly available (https://www.apacrs.org/barrett_universal2). Limitations in terms of AL or target refraction are not published on the website, and it is stated that “The Barrett Universal II Formula is able to predict highly myopic eyes including negative powered IOLs accurately without specialized constants or axial length modification “[11].

However, the tendency for higher MedAEs and the myopic shift we found for Barrett and RBF for minus IOLs has not been described previously, which is most likely due to the uncommonness of myopia of this degree [10, 12, 14].

In 16 patients, we included only one eye in the study, in 21 patients both eyes, which limits our results because of the compounding (correlation) of data with bilateral eyes [16]. As high myopia is uncommon, many previous studies included bilateral cases [3, 21].

Other formulae have shown high accuracy in IOL calculation but were not accessible to us. The Olsen formula, which has also demonstrated good results in highly myopic patients [10], is integrated into the Lenstar (Haag-Streit, Wedel, Germany) and not openly available. The IOLMaster 500 can be updated to offer the Holladay 2 formula, which can be optimized for long ALs by WK adjustment but was inferior to Barrett and Haigis in previous studies [10]. Other formulae that have shown promising results but were not yet considered in this study are the Kane formula (https://www.iolformula.com), [22] the Emmetropia Verifying Optical (EVO) Formula (https://www.evoiolcalculator.com) and the Panacea formula (http://www.panaceaiolandtoriccalculator.com).

To summarize, IOL calculation in highly myopic eyes remains a challenge, even with new formulae at hand. Absolute and refractive errors differ between formulae but also between plus and minus IOLs within a formula. Surgeons should consider these specific alterations in the preoperative planning.

## Data Availability

The manuscript has no associated data in a data repository.

## References

[1] Mamalis N (2000). Complications of foldable intraocular lenses requiring explanation or secondary intervention–1998 survey. J Cataract Refract Surg.

[2] Roessler GF, Dietlein TS, Plange N (2012). Accuracy of intraocular lens power calculation using partial coherence interferometry in patients with high myopia. Ophthalmic Physiol Opt.

[3] Wang L, Shirayama M, Ma XJ, Kohnen T, Koch DD (2011). Optimizing intraocular lens power calculations in eyes with axial lengths above 25.0 mm. J Cataract Refract Surg..

[4] Terzi E, Wang L, Kohnen T (2009). Accuracy of modern intraocular lens power calculation formulas in refractive lens exchange for high myopia and high hyperopia. J Cataract Refract Surg.

[5] Pesudovs K, Garamendi E, Elliott DB (2006). A quality of life comparison of people wearing spectacles or contact lenses or having undergone refractive surgery. J Refract Surg.

[6] Findl O, Drexler W, Menapace R, Heinzl H, Hitzenberger CK, Fercher AF (2001). Improved prediction of intraocular lens power using partial coherence interferometry. J Cataract Refract Surg.

[7] Tehrani M, Krummenauer F, Blom E, Dick HB (2003). Evaluation of the practicality of optical biometry and applanation ultrasound in 253 eyes. J Cataract Refract Surg.

[8] Kohnen S, Brauweiler P (1996). First results of cataract surgery and implantation of negative power intraocular lenses in highly myopic eyes. J Cataract Refract Surg.

[9] Martinez-Enriquez E, Perez-Merino P, Duran-Poveda S, Jimenez-Alfaro I, Marcos S (2018). Estimation of intraocular lens position from full crystalline lens geometry: towards a new generation of intraocular lens power calculation formulas. Sci Rep.

[10] Melles RB, Holladay JT, Chang WJ (2018). Accuracy of intraocular lens calculation formulas. Ophthalmology.

[11] Barrett GD (1993). An improved universal theoretical formula for intraocular lens power prediction. J Cataract Refract Surg.

[12] Roberts TV, Hodge C, Sutton G, Lawless M (2018). Comparison of Hill-radial basis function, Barrett Universal and current third generation formulas for the calculation of intraocular lens power during cataract surgery. Clin Exp Ophthalmol.

[13] Wang JK, Hu CY, Chang SW (2008). Intraocular lens power calculation using the IOLMaster and various formulas in eyes with long axial length. J Cataract Refract Surg.

[14] Chen C, Xu X, Miao Y, Zheng G, Sun Y, Xu X (2015). Accuracy of intraocular lens power formulas involving 148 eyes with long axial lengths: a retrospective chart-review study. J Ophthalmol.

[15] Suto C, Sato C, Shimamura E, Toshida H, Ichikawa K, Hori S (2007). Influence of the signal-to-noise ratio on the accuracy of IOLMaster measurements. J Cataract Refract Surg.

[16] Hoffer KJ, Aramberri J, Haigis W (2015). Protocols for studies of intraocular lens formula accuracy. Am J Ophthalmol.

[17] Norrby S (2008). Sources of error in intraocular lens power calculation. J Cataract Refract Surg.

[18] Petermeier K, Gekeler F, Messias A, Spitzer MS, Haigis W, Szurman P (2009). Intraocular lens power calculation and optimized constants for highly myopic eyes. J Cataract Refract Surg.

[19] Haigis W (2009). Intraocular lens calculation in extreme myopia. J Cataract Refract Surg.

[20] Curtin BJ, Karlin DB (1971). Axial length measurements and fundus changes of the myopic eye. Am J Ophthalmol.

[21] Abulafia A, Barrett GD, Rotenberg M (2015). Intraocular lens power calculation for eyes with an axial length greater than 26.0 mm: comparison of formulas and methods. J Cataract Refract Surg..

[22] Connell BJ, Kane JX (2019). Comparison of the Kane formula with existing formulas for intraocular lens power selection. BMJ Open Ophthalmol.

